# Inhibition of Influenza Virus Replication by Targeting Broad Host Cell Pathways

**DOI:** 10.1371/journal.pone.0110631

**Published:** 2014-10-21

**Authors:** Isabelle Marois, Alexandre Cloutier, Isabelle Meunier, Hana M. Weingartl, André M. Cantin, Martin V. Richter

**Affiliations:** 1 Department of Medicine, Pulmonary Division, Faculty of Medicine and Health Sciences, Université de Sherbrooke, Sherbrooke, Québec, Canada; 2 National Centre for Foreign Animal Disease, Canadian Food Inspection Agency, Winnipeg, Manitoba, Canada, and Department of Medical Microbiology, College of Medicine, University of Manitoba, Winnipeg, Manitoba, Canada; 3 Centre de Recherche du CHUS, Sherbrooke, Québec, Canada; George Mason University, United States of America

## Abstract

Antivirals that are currently used to treat influenza virus infections target components of the virus which can mutate rapidly. Consequently, there has been an increase in the number of resistant strains to one or many antivirals in recent years. Here we compared the antiviral effects of lysosomotropic alkalinizing agents (LAAs) and calcium modulators (CMs), which interfere with crucial events in the influenza virus replication cycle, against avian, swine, and human viruses of different subtypes in MDCK cells. We observed that treatment with LAAs, CMs, or a combination of both, significantly inhibited viral replication. Moreover, the drugs were effective even when they were administered 8 h after infection. Finally, analysis of the expression of viral acidic polymerase (PA) revealed that both drugs classes interfered with early events in the viral replication cycle. This study demonstrates that targeting broad host cellular pathways can be an efficient strategy to inhibit influenza replication. Furthermore, it provides an interesting avenue for drug development where resistance by the virus might be reduced since the virus is not targeted directly.

## Introduction

Influenza A viruses (IAV) cause acute respiratory tract infections that are generally mild but that can also lead to severe lung pathology, respiratory distress, and death [1]. In addition to seasonal outbreaks which have major global health, social and economic impacts, IAV bear the potential to develop into new pandemic strains, as highlighted by the frequent emergence of H5N1 viruses, the new H7N9 virus, and the 2009 H1N1 pandemic virus (A(H1N1)pdm09) [2]–[4]. Even though vaccination is the best strategy to protect against infection, the generation of vaccines against seasonal IAV is a time-consuming process required annually and the emergence of pandemic IAV represents an additional challenge in terms of vaccine development and availability.

There are currently two classes of antivirals available to treat IAV infection that target either the M2 ion channel (adamantanes) or viral neuraminidase (oseltamivir, zanamivir, peramivir and laninamivir) [5], [6]. However, because of the widespread resistance in circulating strains, adamantanes are rarely used today [7], [8]. Furthermore, resistance to neuraminidase inhibitors is continuously reported in newly emerging influenza viruses [5]. For instance, oseltamivir resistance was widespread among seasonal H1N1 strains in the 2008–2009 season [9]. Although the overall proportion of resistant isolates is relatively low among the A(H1N1)pdm09 virus, resistant isolates of the A(H1N1)pdm09 virus are continuously reported, and the proportion of drug-resistant cases not associated with oseltamivir exposure has increased significantly in US (74% in 2010–2011) [10], [11]. Consequently, there is a need to develop new antiviral strategies to overcome resistance.

IAV, like other viruses, require the host cell machinery to produce infectious progeny viruses. Targeting the host components essential for infection and replication therefore constitutes a promising antiviral strategy that may circumvent antiviral drug resistance [12]–[15]. One of these targets is endosomal acidification, which represents a critical step for IAV entry into cells [16], [17]. The decreased pH within endosome induces conformational changes in IAV hemagglutinin (HA) to expose the fusion peptide, thereby allowing fusion between the viral envelope and the endosome [18]. Indeed, inhibition of the V-type ATPases with the antibiotic bafilomycin A was reported to inhibit influenza A and B replication [19], [20]. Moreover, studies with the anti-malaria drug chloroquine, a weak base that inhibits endosomal acidification, have demonstrated its antiviral effects against influenza A and B viruses, Chikunguya virus, and the human immunodeficiency virus [21]–[24]. In addition, chloroquine also inhibits low pH-dependent proteases in the Golgi network that participate in the maturation of nascent viral proteins [25], [26].

Host calcium-dependent proteins also represent an interesting target, as many calcium-dependent proteins have been shown to participate in the IAV replication cycle, such as cellular PKCβII which is involved in IAV morphogenesis and ribonucleoprotein (RNP) import by regulating PKCα activity. Moreover, calnexin and calreticulin promote folding, prevent premature oxidation and oligomerization, suppress degradation of HA, and are important for efficient maturation of viral neuraminidase [27]–[32]. Consequently, drugs that affect intracellular calcium concentrations, such as verapamil, interfere with virus assembly and budding [31].

In this study, we compared the efficacy of commercially available drugs that either modulate endosomal pH or intracellular calcium concentration to interfere with IAV replication. The drugs were tested alone or in combination in MDCK cells against human, avian, and swine viruses from different subtypes.

## Materials and Methods

### Chemicals

Oseltamivir carboxylate was purchased from MedChemexpress CO., Ltd, (Monmouth Junction, NJ). Lysosomotrophic agents amodiaquin dihydrochloride dihydrate, bafilomycin A from *Streptomyces griseus*, chloroquine diphosphate salt, quinacrine dihydrochloride, quinidine anhydrous, mefloquine hydrochloride, and primaquine diphosphate were purchased from Sigma-Aldrich (St-Louis, MO). Quinine sulfate was obtained from Fisher (Fair Lawn, NJ). Calcium modulators calcimycin (A23187), capsaicin, 5-(N,N-Dimethyl) amiloride hydrochloride, and verapamil hydrochoride were purchased from Sigma-Aldrich whereas TMB-8 hydrochloride was purchased from Calbiochem (Mississauga, ON). Mefloquine, calcimycin, bafilomycin A, and capsaicin were dissolved in DMSO (Sigma-Aldrich) in order to have a final solvent concentration of less than 0.1%, whereas other compounds were dissolved in culture medium. At this concentration, DMSO showed no apparent toxicity in MDCK cells (less than 1%, data not shown).

### Cell culture

Madin-Darby Canine Kidney cells (MDCK) were obtained from the American Type Culture Collection (CCL-34) and maintained in Minimal Essential Media (EMEM; Wisent, Canada) supplemented with 10% FBS, 100 µg/ml streptomycin, 100 U/ml penicillin, 1 mM sodium pyruvate, 100 µM nonessential amino acids, and 2 mM L-glutamine (Wisent).

### Viruses

The mouse-adapted influenza H3N2 A/Hong Kong/X-31 (X-31) and H1N1 A/Puerto Rico/8/34 (PR8) viruses were kindly provided by Dr. David Topham (University of Rochester Medical Center, USA) and amplified in 10-day-old embryonated hens’ eggs using standard procedures [33]. The human isolate H1N1 A/California-like/2009 (A/H1N1/2009) was isolated in Montreal, Canada, during the 2009 pandemic and obtained from the Laboratoire de Santé publique du Québec. The virus was amplified in MDCK cells. The avian strains H5N2 A/Teal/Germany/WV632/2005 (TG05) and H5N1 A/Domestic goose/Germany/R1400/2007 (R1400) were a kind gift of Dr. Timm Harder (Friedrich Loeffler Institute, Germany). The avian strain H5N2 A/Emu/Texas/39924/1993 (Emu-Tx) and the swine strain H1N1 A/Swine/Alberta/OTH-33-2/2009 (OTH-33-2) were from archives of the National Centre for Foreign Animal Disease (NCFAD) (Canadian Food Inspection Agency, Winnipeg, Canada). Avian and swine influenza strains were expanded in embryonated hens’ eggs. All experiments involving the avian and swine viruses were performed at NCFAD.

### Viral plaque inhibition assays

To determine the antiviral effect of the compounds for X-31, PR8, and A/H1N1/2009 viruses, viral plaque inhibition assays were performed as previously described [34], [35]. Briefly, confluent MDCK cells seeded in a 24-well plate were washed with phosphate-buffered saline (PBS; Wisent) and treated for 1 h with various concentrations of each compound or the vehicle, as a negative control, diluted in EMEM containing 0.1% bovine serum albumin (incomplete medium; iEMEM). Cells were then infected with 25 plaque-forming units (PFU) of the different viruses in the medium containing the compounds for 1 h. Cells were washed once with PBS and medium was replaced with a mix of sterile Avicel 1.8% containing 1 µg/ml TPCK-treated trypsin (Sigma) (Avicel 3.6% diluted 1∶1 with 2X iEMEM) and the compound dilutions. After 48 h at 37°C 5% CO_2_, cells were washed twice with PBS and fixed with Carnoy fixative (methanol: acetic acid, 3∶1) for 20 min at 4°C. Viral plaques were revealed by crystal violet staining (1% solution in 20% methanol/water). For the Emu-Tx, OTH-33-2, TG05, and R1400 viruses, only the effective doses of the compounds identified with the mouse-adapted and human viruses were tested. The viral plaques were revealed using an immunostaining assay. Briefly, acetone-fixed cells were incubated with an anti-influenza A NP-28 monoclonal antibody (provided by Dr. Yang, NCFAD), followed by a peroxidase-labeled anti-mouse IgG antibody (Jackson ImmunoResearch Laboratories). Positive cells were visualised using True Blue Peroxidase Substrate (KPL) or 3-amino-9-ethyl-carbazole AEC Substrate (Sigma).

### RNA extraction and viral quantification by quantitative real-time RT-PCR (qPCR) analysis

MDCK cells seeded in a 6-well plate were treated for 1 h with the different compounds and infected with the PR8 virus at an MOI of 1. After 1 h, cells were washed with PBS and incubated in iEMEM supplemented with the compounds. At 1 and 4 h post-infection (p.i.), cells were lysed in TRIzol (Invitrogen). RNA was extracted following the manufacturer’s instructions. To assess the influenza PA expression, RNA was reverse-transcribed with the Omniscript kit (Qiagen) using random decamers (Austin, TX, USA) and qPCR assays were performed with the Quantitect SYBR Green PCR kit (Qiagen) using a Rotor Gene-6000 cycler (Corbett-Research) as previously described [34], [35]. The relative expression of PA was determined with the 2^−ΔΔCt^ method using the 18S rRNA expression for normalization as previously described [35], [36]. Conversion to percentages was done by setting the value obtained in the infected and untreated cells by the 2^−ΔΔCt^ method to 100%, i.e. the maximal level of viral PA gene expression achievable in infected cells.

### Cytotoxicity assays

For cytoxicity assays, MDCK cells were seeded (5×10^4^ MDCK cells/well in 96-well culture plates) and incubated at 37°C in complete medium for 24 h before drug treatment. Cells were cultured for 2 days with each compound at various concentrations (0,001 nM-3 mM). At the end of the incubation period, cell viability was assessed by the XTT (2,3-bis-(2-methoxy-4- nitro-5-sulfophenyl)-2 H-tetrazolium-5-carboxanilide) assay (Invitrogen, Carlsbad, CA, USA). The spectrophotometric absorbance of the samples was measured using a microplate reader (Thermomax microplate reader, Molecular Devices, Sunnyvale, CA, USA) at 450 nm with a reference wavelength of 690 nm. Each measurement was performed in triplicate and the data reported were mean values of at least 2 experiments. Cell viability (%) was calculated according to the following equation: Cell viability (%) = (A_λ_450 of treated wells/A_λ_450 of control wells) × 100. CC_50_ values were calculated using nonlinear regression curve fit with a variable slope using GraphPad Prism 6.0 software (GraphPad Software Inc., La Jolla, CA).

### Statistical analysis

The statistical analyses were performed using GraphPad Prism 6.0 software. Raw data were analyzed by one-way ANOVA followed by Bonferroni’s or Dunnett's a posteriori tests depending on the experimental context to determine significant differences between conditions. For dose-response curves, inter-experimental data were normalized in the form of percentages of maximum. The percentage values were then transformed into arcsin values before being analyzed with one-way ANOVA followed by the appropriate post-hoc test. A *p* value <0.05 was considered statistically significant (*p<0.05; **p<0.01;***p<0.001). EC_50_ and CC_50_ were calculated using nonlinear regression dose-response inhibition.

## Results and Discussion

### Effects of lysosomotropic agents on influenza A virus replication

**Figure 1:**
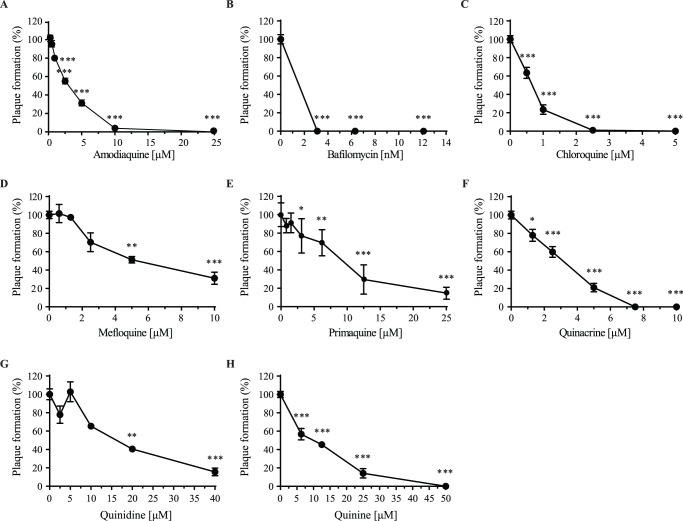
Antiviral effects of lysosomotropic alkalinizing agents on influenza replication. MDCK cells were treated with various concentrations of (A) amodiaquine, (B) bafilomycin, (C) chloroquine, (D) mefloquine, (E) primaquine, (F) quinacrine, (G) quinidine or (H) quinine 1 h before infection and after infection for 48 h with 25 PFU of the PR8 virus. Viral plaques were counted and results are expressed as a percentage of plaque formation compared to untreated cells (Plaque formation (%)). Statistical significance: p<0.05, **p<0.01, ***p<0.001. Results of three to six independent experiments, performed in triplicate are shown.

We first compared the efficacy of novel LAAs to interfere with IAV replication in a viral plaque inhibition assay in MDCK cells which provides a direct evaluation of ability of compounds to block viral plaque formation. In agreement with previous studies [20], [26], bafilomycin A and chloroquine completely inhibited the replication of PR8, X-31, and A/H1N1/2009 in the low micromolar range (**Fig. 1** **B–C,** **Table 1**). Moreover, all the other LAAs were also able to significantly interfere with PR8 replication in a dose-dependent manner (**Fig. 1** **A, D–H**). All LAAs inhibited IAV replication at a dose range below 10 µM, except quinidine, which had to be used at least at 16.2 µM **(** **Fig. 1**). At the lowest concentrations giving rise to maximal inhibition of PR8 viral plaque formation, all LAAs tested showed no cytotoxicity except for quinacrine (10% cell death at 6.5 µM but no toxicity at 4 µM; Figure 1 and Table 2, and data not shown). Furthermore, even though quinine had a higher EC_50_ than mefloquine, it was able to completely abrogate PR8 replication at a higher concentration, which was not the case for mefloquine. In general, compared to chloroquine, amodiaquine and quinacrine showed similar antiviral activities against influenza viruses. Interestingly amodiaquine was the most active against X-31, chloroquine was the most potent against PR8 whereas amodiaquine and quinacrine were more effective against the A(H1N1)pdm09 virus. Overall, among the new compounds tested, we demonstrated that amodiaquine is the most potent, followed by quinacrine, mefloquine, quinine, quinidine, and primaquine. In addition to its antiviral activity determined in viral plaque formation inhibition assay, amodiaquine also inhibited viral replication in growth kinetics assays (Figure S1). Moreover, additional experiments with amodiaquine were performed in a human bronchial epithelial cell line (Calu-3) that represents a more physiologically relevant primary target for influenza virus infection. Interestingly, the results obtained with this cell line matched those obtained in MDCK cells. Indeed, in Calu-3 cells, the EC_50_ of amodiaquine against PR8 is between 1.25–2.50 µM compared to an EC_50_ of 2.76 µM in MDCK cells (Table S1).

**Table 1:** Antiviral activities of the compounds tested in this study against different strains of influenza viruses.

Compound	EC_50_ values (µM)^a^		
	A/Hong Kong/X31 (H3N2)	A/Puerto Rico/8/34 (H1N1)	A/California-like/2009 (H1N1)
**Lysosomotropic alkalinizing agents**			
Amodiaquine	2.63±0.90	2.76±0.95	1.48±0.92
Bafilomycin A	4.00^b^	4.00^b^	INH^c^
Chloroquine	4.31±0.96	0.61±0.91	4.01±0.80
Mefloquine	6.66±0.75	5.13±0.87	N/I^d^
Primaquine	29.47±9.42	8.58±0.89	28.55±9.45
Quinacrine	5.97±0.91	2.96±0.87	1.88±0.87
Quinidine	19.83±8.83	16.21±8.90	5.11±0.76
Quinine	17.13±8.70	9.10±0.89	12.19±8.49
**Calcium modulators**			
A23187 (calcimycin)	0.08±0.01	0.08±0.01	0.22±0.01
5-(N,N-Dimethyl)amiloride hydrochloride	20.38±8.62	31.08±7.72	63.51±5.77
Capsaicin	55.17±7.20	58.79±8.32	44.69±8.15
TMB-8	21.69±9.31	2.50±0.91	12.71±8.65
Verapamil	19.62±8.75	4.91±0.84	26.35±8.69
**Neuraminidase inhibitor**			
Oseltamivir carboxylate	0.74±0.08^b^	49.92±8.42^b^	102.90±32.12^b^

a The required concentration to reduce 50% of plaque formation (EC_50_) was calculated by a nonlinear regression dose-response inhibition.

b [ ] in nM.

c INH: 100% inhibition at the smallest concentration tested.

d N/I: no inhibition observed before complete cytotoxicity by the compound.

**Table 2:** Cytotoxicity of LAAs and CMs in MDCK cells.

Compound	CC_50_ (µM)^a^	*In vitro* therapeutic index^b^
**Lysosomotropic alkalinizing agents**		
Amodiaquine	26.24±8.43	20.03
Bafilomycin	16.77±9.02^c^	4.19
Chloroquine	31.94±8.28	52.36
Mefloquine	22.94±1.30	4.47
Primaquine	126.10±80.5	14,70
Quinacrine	14.58±8.51	4.93
Quinidine	167.30±94.21	10.32
Quinine	408.60±82.24	44.90
**Calcium modulators**		
Capsaicin	156.90±9.63	2.67
A23187 (calcimycin)	0.413±0.21	5.16
TMB-8	52.27±8.38	20.91
Verapamil	280.40±80.09	57.11
5-(N,N-Dimethyl) amiloride hydrochloride	175.00±10.24	5.63
**Neuraminidase inhibitor**		
Oseltamivir carboxylate	>1000.00^d^	>20.03

a Cytotoxicity assessed by XTT cell viability assay. Drug concentrations reducing cell viability by 50% (CC_50_) were determined from dose-response curves.

b *In vitro* therapeutic index calculated with the EC_50_ against PR8 virus replication.

c [ ] in nM.

d No cell death was detected at concentration up to 1 mM.

Interestingly, the viral strains differed in their sensitivity to the different compounds. While mefloquine efficiently inhibited the replication of the PR8 and X-31 viruses, it was unable to prevent A/H1N1/2009 replication. Moreover, quinidine was more efficient at decreasing A/H1N1/2009 replication whereas primaquine, quinine, and chloroquine were less efficient against X-31 and, to a lesser extent, to A/H1N1/2009 viruses. In summary, these experiments demonstrate that viral strains differ in their sensitivity to LAAs, PR8 being the most sensitive, followed by A/H1N1/2009, and X-31. This is in accordance with a study that demonstrated that H1N1 viruses are more sensitive to chloroquine than H3N2 viruses due to differences in the electrostatic potential of HA2 [37].

### Antiviral efficacy of calcium modulators on influenza replication

**Figure 2:**
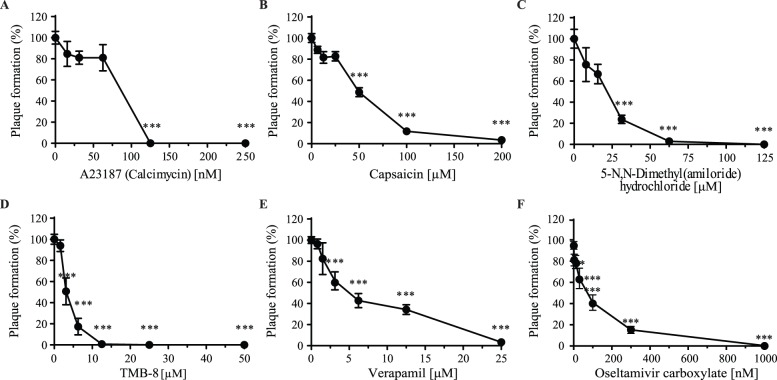
Antiviral effects of calcium modulators and oseltamivir on influenza replication. MDCK cells were treated with various concentrations of (A) calcimycin (A23187), (B) verapamil, (C) capsaicin, (D) TMB-8, (E) 5-(N,N-dimethyl)amiloride hydrochloride or (F) oseltamivir carboxylate as previously described in Figure 1 and the antiviral effect was tested against the PR8 virus. Viral plaques were counted and results are expressed as a percentage of plaque formation compared to untreated cells (Plaque formation (%)). Statistical significance: p<0.05, **p<0.01, ***p<0.001. Results of three to six independent experiments, performed in triplicate are shown.

In order to target intracellular calcium concentrations, we evaluated the efficacy of verapamil, a Ca^2+^ channel inhibitor, as well as calcimycin (A23187), a calcium-specific ionophore, to prevent IAV replication. In accordance with previous studies, our results demonstrated that both compounds inhibited viral replication in a dose-dependent manner, calcimycin being the most efficient but having cytotoxic effect at relatively low concentration (**Fig. 2A, E** **and** **Table 1** **–** **2**) [31], [38]. We next evaluated the efficacy of other CMs that had never been characterized in their capacity to interfere with IAV replication. We observed that capsaicin, a modulator of voltage-activated calcium channel [39], TMB-8, an ER Ca^2+^ store release inhibitor [40], inhibited viral replication in a dose-dependent manner (**Fig. 2**). 5-(N,N-Dimethyl)amiloride hydrochloride, a selective blocker of Na^+^/H^+^ antiport that also possess activity on Ca^2+^ exchange, also inhibited viral replication [41], Interestingly, TMB-8 was as effective as verapamil against PR8 and X-31 but was more potent against the A(H1N1)pdm09 virus (**Table 1**). At the lowest concentrations giving rise to maximal inhibition of PR8 viral plaque formation, all CMs tested showed no cytotoxicity except for capsaicin and TMB-8. Indeed, capsaicin induced 50% cell death at 200 µM but had no significant toxicity at 100 µM (Figure 1 and Table 2, and data not shown) and TMB-8 induced 25% cell death at 25 µM but at 10 µM, a concentration that inhibited 90% of PR8 replication, no significant toxicity was observed (Figure 1 and Table 2, and data not shown). We also performed experiments with verapamil in Calu-3 cells and the results obtained also matched those obtained in MDCK cells. Indeed, in Calu-3 cells, the EC_50_ of verapamil against PR8 is between 1.25–2.50 µM compared to an EC_50_ of 4.91 µM in MDCK cells (Table S1).

As we previously observed with the LAAs, the sensitivity to the different CMs varied between the strains without clear correlation. Overall, these results support the literature showing that the level of intracellular calcium is crucial for influenza virus replication and here, we described novel CMs that could potentially be used to block IAV replication.

### The combination of lysosomotropic alkalinizing agents and calcium modulators show increased inhibitory effect on influenza replication

**Figure 3:**
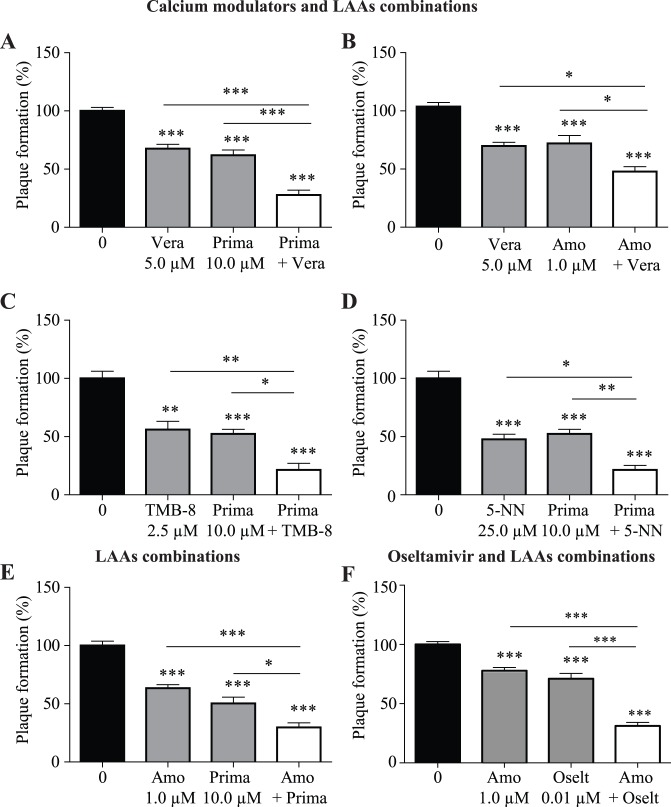
Antiviral effects of the combination of different treatments on influenza replication. MDCK cells were treated as described in Figure 1. The combinations of calcium modulators with lysosomotropic alkalinizing agents was tested against the PR8 virus. Combination of a calcium modulator with a lysosomotropic alkalinizing agents (A, B, C, D), combination of two lysosomotropic alkalinizing agents (E), and combination of amodiaquine and oseltamivir carboxylate (F). Viral plaques were counted and results are expressed as a percentage of plaque formation compared to untreated cells (Plaque formation (%)). Statistical significance: p<0.05, **p<0.01, ***p<0.001. Results of three to six independent experiments, performed in triplicate are shown. Abbreviations used: Amodiaquine (Amo); 5-(N,N-Dimethyl)amiloride hydrochloride (5-N,N); Oseltamivir (Oselt); Primaquine (Prima); TMB-8 hydrochloride (TMB-8); Verapamil (Vera).

Since LAAs and CMs were able to interfere with viral replication by targeting different cellular pathways, we next sought to determine if the combination of both classes of compounds using low doses could improve the antiviral effect. All of the combinations of a LAA with a CM tested were more effective than the use of single compounds alone (**Fig. 3A–D**). Of note, all drug combinations tested did not lead to any cytotoxic effect (data not shown). The combinations of verapamil with primaquine or amodiaquine show an increased inhibitory effect compared to each compound used alone. Interestingly, the combination of the CMs (TMB-8 or 5-(N,N-dimethyl)amiloride hydrochloride) with the LAA primaquine were the most efficient, yielding to approximately 79% inhibition of viral replication at low doses. The combination of amodiaquine and primaquine, two LAAs, also led to an increased inhibition of viral replication which suggests that these 2 compounds might act on different components of the early entry process of the virus (**Fig. 3E**).

We next sought to investigate whether we could improve the efficiency of oseltamivir carboxylate by combining it to amodiaquine, a drug currently used to treat malaria infection. Treatment of cells with 0.01 µM of oseltamivir or 1.0 µM of amodiaquine led to approximately 30% and 26% of reduction of PR8 replication, respectively (**Fig.** **3F** **)**. Strikingly, the combination of both compounds strongly impeded viral replication (70% inhibition). A similar effect was also observed for the A/H1N1/2009 virus (data not shown). Thus, these results show that oseltamivir carboxylate could be used in combination with a LAA to improve its efficiency. Even though Tamiflu® is the most commonly used antiviral against influenza, many side effects have been reported [42]. Although in vivo studies are needed, our results suggest that a treatment combining lower doses of oseltamivir and LAAs might allow the use of oseltamivir at lower doses and thus, possibly reduces its side effects.

### Lysosomotropic alkalinizing agents and calcium modulators block early events of influenza virus replication cycle

**Figure 4:**
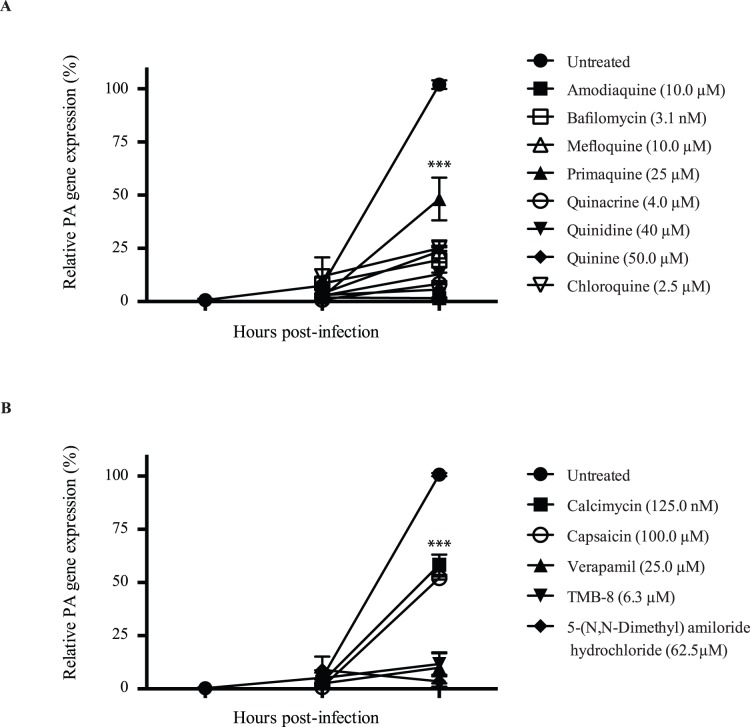
Expression of influenza PA gene following treatment of cells with the antiviral agents. MDCK cells were treated with (A) lysosomotropic alkalinizing agents or (B) calcium modulators for 1 h before and after infection with the PR8 virus at an MOI of 1. Cells were harvested at the indicated time points, total RNA was extracted, reverse transcribed, and PA expression was quantified by semi-quantitative real-time PCR. The relative PA gene expression is expressed as a percentage of the untreated and infected control. Results of two independent experiments, performed in duplicate are shown. Statistical significance : for all the compounds compared to infected and untreated cells ***p<0.001.

To determine at which step in the replication cycle the drugs were interfering, we treated MDCK cells with LAAs or CMs 1 h before infection and during the course of the experiment at the lowest dose that provided maximal inhibition and that did not induce significant cytotoxicity (**Fig. 1** **–** **2** **and** **Table 2**). Cells were infected with the PR8 virus at an MOI of 1 and RNA expression of PA was quantified by qPCR at 1 and 4 h p.i. No significant increase in PA expression was observed at 1 h p.i., consistent with previous studies [43], but was significantly increased at 4 h p.i. in untreated cells, as the virus began to replicate (**Fig**. **4A**). In contrast, treatment of cells with bafilomycin, quinine, quinacrine, and quinidine was associated with a decrease of more than 90% of PA expression, whereas mefloquine and amodiaquine repressed 82% of gene expression. Primaquine was the least efficient but was still able to inhibit 62% of PA expression. As indicated by the decrease in PA accumulation, our results suggest that these compounds interfere with early events of the influenza life cycle, and according to the literature, most likely inhibition of viral fusion [21], [37], [44], [45].

We next performed similar experiments with CMs. Treatment of cells with verapamil, TMB-8, and 5-(N, N-dimethyl)amiloride hydrochloride almost completely abolished PA gene expression (**Fig**. **4B**). As previously mentioned, calcium-dependent proteins are important for promoting the endocytic pathway, RNP import, and viral RNA transcription [27]–[31], [46]. CMs therefore most likely interfere with early events in IAV replication cycle. Surprisingly, calcimycin, which had the lowest EC_50_ (**Table 1**) was the least efficient in this assay, yielding only 42% inhibition of PA expression. Capsaicin was also associated with a decrease of approximately 48% of PA expression. This suggests that calcimycin and capsaicin interfere mostly with later steps of IAV replication. For instance, it has been shown that the calcium-ionophore A23187 inhibited proteolytic cleavage of HA0 into HA1 and HA2 fragments [38]. Indeed, the activation of HA requires host enzymes such as proprotein convertases that need the presence of calcium to cleave the target sequence [46], [47]. Calcimycin and capsaicin might therefore interfere with the cleavage of HA0, which occurs later during the replication cycle.

### Antiviral effects of lysosomotropic alkalinizing agents on avian viruses and on a swine virus from the 2009 H1N1 pandemic

**Figure 5:**
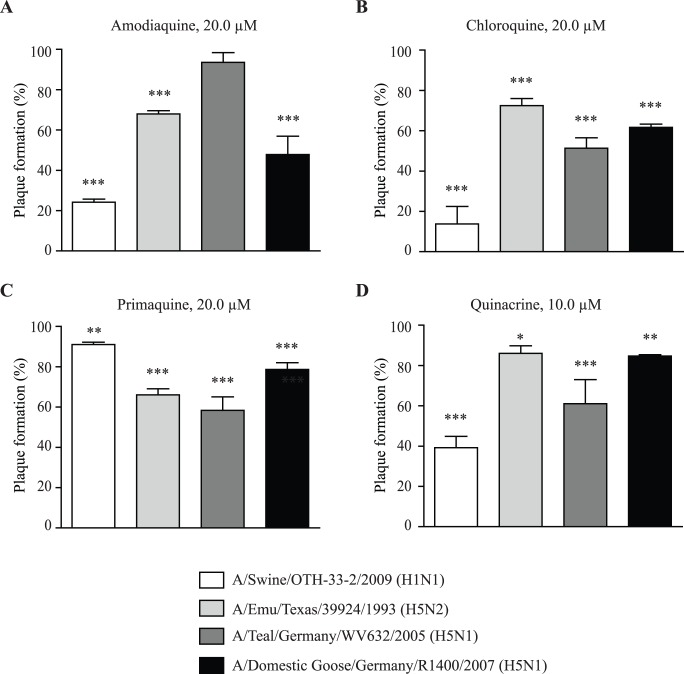
Antiviral effects of lysosomotropic alkalinizing agents on the replication of avian and swine isolates. MDCK cells were treated as described in Figure 1 with (A) amodiaquine, (B) chloroquine, (C) primaquine or (D) quinacrine and infected with 25 PFU of the H1N1 swine isolate A/Swine/OTH-33-2/2009 or the avian isolates H5N2 A/Emu/Texas/39924/1993, H5N1 A/Teal/Germany/WV632/2005, and H5N1 A/Domestic goose/Germany/R1400/2007. Viral plaques were counted and results are expressed as a percentage of plaque formation compared to its respective control i.e. untreated cells infected with the corresponding influenza strain (Plaque formation (%)). Results are representative of three to six independent experiments. Statistical significance: **p<0.01, ***p<0.001.

To determine whether the antiviral effects of LAAs could be observed with avian strains and a swine strain isolate of the 2009 pandemic influenza, plaque inhibition assays were carried out with the highest non-toxic concentration of the various compounds. For the swine strain (A/Swine/OTH-33-2/2009 (H1N1)), chloroquine, amodiaquine and quinacrine were most effective in inhibiting replication (more than 60% inhibition) compared to primaquine (less than 20% inhibition) (**Figure 5**). At equivalent concentrations of LAAs, the swine OTH-33-2 isolate and the human pH1N1/2009 (**Figure 5** **and** **Table 1**) seemed to have comparable sensitivities although the swine isolate was slightly less sensitive. This might be attributable to possible genetic variations between isolates. Interestingly, amodiaquine was the most potent compound at inhibiting the replication of the highly pathogenic avian influenza H5N1 (HPAI; A/Domestic Goose/Germany/R1400/2007 (H5N1)) rather than the low pathogenic avian influenza strain (LPAI; A/Teal/Germany/WV632/2005 (H5N1)) (**Figure 5A**). In contrast, chloroquine, primaquine and quinacrine seem more effective at inhibiting the low pathogenic avian influenza stain rather than the highly pathogenic (**Figure 5B–D**). All LAAs were only capable of inhibiting Emu-Tx avian strain (A/Emu/Texas/39924/1993 (H5N2)) replication by less than 40% (**Figure 5**). Overall, we demonstrated that the swine isolate (H1 subtype) appears to be generally more sensitive to inhibition of endosomal acidification induced by LAAs than the avian strains (H5 subtype), except for the primaquine. Again, in accordance with the study by Di Trani et al. that used only chloroquine, LAAs were less effective against human than avian strains suggesting that endosomal pH dependence is more associated with human viral adaptation than avian viruses [37]. Taken together, our observations support the evidence that a lower pH is necessary for influenza replication and that targeting endosomal acidification in host cells can be considered as a potential avenue for inhibiting replication of different influenza strain.

### Efficacy of lysosomotropic alkalinizing agents and calcium modulators in post-infection treatment

**Figure 6:**
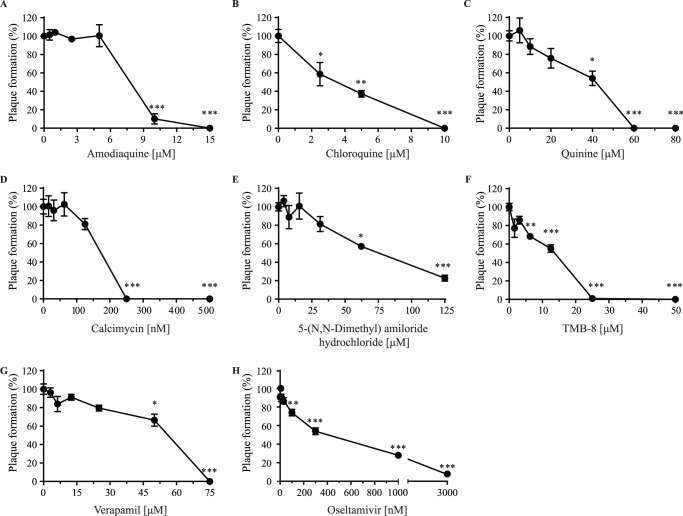
Efficacy of antivirals 8 h after infection. MDCK cells were infected with 25 PFU of the PR8 virus and 8 h later, (A) amodiaquine, (B) chloroquine, (C) quinine, (D) calcimycin (E) 5-(N,N-Dimethyl)amiloride hydrochloride, (F) TMB-8, (G) verapamil or (H) oseltamivir carboxylate, were added to the culture media for 48 h. Viral plaques were counted and results are expressed as a percentage of plaque formation compared to untreated cells (Plaque formation (%)). Statistical significance: p<0.05, **p<0.01, ***p<0.001. Results of two independent experiments performed in duplicate are shown.

To determine if the compounds could be used not only in prophylaxis but also as a treatment, MDCK cells were infected with the PR8 virus and treated 8 h post-infection with LAAs or CMs, a time-point at which full initiation of the viral life cycle is in place. The LAAs amodiaquine, chloroquine, and quinine were all able to abrogate viral replication although the concentrations needed to achieve this effect were twice those used in prophylaxis, except quinine which required only a slight increase in concentration (60 µM instead of 50 µM) (**Fig. 6**). Similarly, the CMs calcimycin, TMB-8, and verapamil were also able to completely inhibit viral replication with concentrations double those of prophylaxis. Lastly, treatment with 5-(N,N-dimethyl)amiloride hydrochloride led to 80% inhibition of viral replication at the highest dose. In comparison, the concentration of oseltamivir needed to achieve a 50% reduction of the influenza replication was 6 times higher as a treatment than in prophylaxis (EC_50_ of 313 nM vs 50 nM, respectively). Thus, these results demonstrate that LAAs and CMs could be used in prophylaxis as well as a treatment to reduce viral replication.

## Conclusions

This study demonstrates that drugs targeting broad host cell pathways available on the market today to treat other diseases, can inhibit influenza A virus replication pre- and post- infection, suggesting that they may potentially be used for prophylaxis as well as post-exposure treatment. More specifically, the modulation of endosomal acidification as well as intracellular calcium levels significantly impaired viral replication at concentrations below those that show little cytotoxicity. Furthermore, we identified new endosomal and calcium modulators that have potential as antivirals against influenza viruses. The demonstration of the efficacy of LAAs and CMs against influenza and the knowledge of their respective physico-chemical properties thereby represent interesting avenues for the development of new antivirals against influenza. Further studies performed in animal models should be done to determine the pharmacodynamics, pharmacokinetics, and how the different compounds reach a therapeutic level in the lungs.

## Supporting Information

Figure S1 Replication growth kinetics of the PR8 influenza virus in MDCK cells upon amodiaquine treatment. Multiple-cycle growth curves were obtained by infecting MDCK cells plated in 24-well plate with 25 PFU (MOI = 0.0001) in the presence or the absence of amodiaquine (1.25–25 µM). Viruses in the supernatant were titrated in MDCK cells and expressed as PFU/ml at the indicated time post-infection. Each point represents the mean PFU/ml ± SEM from 2 experi- ments. *** p<0.001 compared to value for untreated cells at corresponding time point.
(EPS)

Table S1 **Antiviral activities of the selected compounds tested in Calu-3 cells against the A/Puerto Rico/8/34 (H1N1) influenza virus.**
(DOCX)
