# Supplementary material for: Inhibition of Influenza Virus Replication by Targeting Broad Host Cell Pathways
Source: PLoS One. 2014 Oct 21;9(10):e110631. doi: 10.1371/journal.pone.0110631 (PMC4204995; doi:10.1371/journal.pone.0110631)
Supplement: Table S1 — Antiviral activities of the selected compounds tested in Calu-3 cells against the A/Puerto Rico/8/34 (H1N1) influenza virus. (DOCX) [file pone.0110631.s002.docx]

| **Compounds** | **EC_50_ values (µM)** ^a^ | **CC_50_ values (µM)** ^b^ |
| --- | --- | --- |
| Amodiaquine | 1.25-2.50^c^ | 53.57 ± 8.80 |
| Verapamil | 1.25-2.50^c^ | 225.6 ± 88.90 |

**Table S1. Antiviral activities of the selected compounds tested in Calu-3 cells against the A/Puerto Rico/8/34 (H1N1) influenza virus.**

^a^ The required concentration to reduce 50 % of plaque formation (EC_50_) was calculated by a nonlinear regression dose-response inhibition. Briefly, Calu-3 cells were exposed to 25 PFU of the PR8 virus in the presence of various concentrations of the drugs (1.25-25 µM). Supernatants were recovered 48 h p.i. and viral titers were determined using viral plaque assay in MDCK cells.

^b^ The concentration that causes 50 % of cell death (CC_50_) was calculated by a nonlinear regression dose-response curve using the XTT cytotoxic assay.

^c^ Estimated EC_50_ based on the lowest concentrations tested.
